# 2-(6,7-Dimethyl-3-methyl­sulfanyl-1-benzofuran-2-yl)acetic acid

**DOI:** 10.1107/S1600536808024288

**Published:** 2008-08-06

**Authors:** Hong Dae Choi, Pil Ja Seo, Byeng Wha Son, Uk Lee

**Affiliations:** a Department of Chemistry, Dongeui University, San 24 Kaya-dong, Busanjin-gu, Busan 614-714, Republic of Korea; b Department of Chemistry, Pukyong National University, 599-1 Daeyeon 3-dong, Nam-gu, Busan 608-737, Republic of Korea

## Abstract

In the title compound, C_13_H_14_O_3_S, the methyl group of the methyl­sulfanyl substituent is almost perpendicular to the plane of the benzofuran fragment [80.5 (9)°]. The carboxylic acid groups are involved in inter­molecular O—H⋯O hydrogen bonds, which link the mol­ecules into centrosymmetric dimers. These dimers are further packed into stacks along the *a* axis by C—H⋯π inter­actions.

## Related literature

For related structures, see: Choi *et al.* (2007 ▶); Seo *et al.* (2007 ▶).
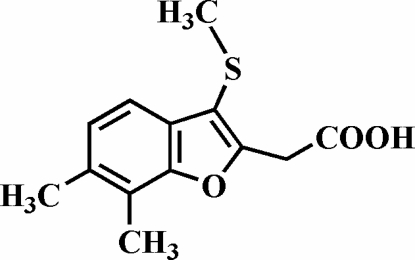


## Experimental

### 

#### Crystal data

C_13_H_14_O_3_S
*M* *_r_* = 250.30
Monoclinic, 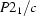

*a* = 18.050 (2) Å
*b* = 4.9422 (5) Å
*c* = 13.885 (1) Å
β = 104.451 (2)°
*V* = 1199.4 (2) Å^3^
*Z* = 4
Mo *K*α radiation
μ = 0.26 mm^−1^
*T* = 173 (2) K
0.40 × 0.20 × 0.10 mm

#### Data collection

Bruker SMART CCD diffractometer
Absorption correction: none
6673 measured reflections
2595 independent reflections
2269 reflections with *I* > 2σ(*I*)
*R*_int_ = 0.035

#### Refinement

*R*[*F* ^2^ > 2σ(*F* ^2^)] = 0.038
*wR*(*F* ^2^) = 0.119
*S* = 1.17
2595 reflections
161 parameters
H atoms treated by a mixture of independent and constrained refinement
Δρ_max_ = 0.42 e Å^−3^
Δρ_min_ = −0.25 e Å^−3^

### 

Data collection: *SMART* (Bruker, 2001 ▶); cell refinement: *SAINT* (Bruker, 2001 ▶); data reduction: *SAINT*; program(s) used to solve structure: *SHELXS97* (Sheldrick, 2008 ▶); program(s) used to refine structure: *SHELXL97* (Sheldrick, 2008 ▶); molecular graphics: *ORTEP-3* (Farrugia, 1997 ▶) and *DIAMOND* (Brandenburg, 1998 ▶); software used to prepare material for publication: *SHELXL97*.

## Supplementary Material

Crystal structure: contains datablocks global, I. DOI: 10.1107/S1600536808024288/hb2769sup1.cif

Structure factors: contains datablocks I. DOI: 10.1107/S1600536808024288/hb2769Isup2.hkl

Additional supplementary materials:  crystallographic information; 3D view; checkCIF report

## Figures and Tables

**Table 1:** Hydrogen-bond geometry (Å, °)

*D*—H⋯*A*	*D*—H	H⋯*A*	*D*⋯*A*	*D*—H⋯*A*
C9—H9*A*⋯*Cg*^i^	0.98	2.86	3.617 (2)	135
O2—H2*O*⋯O3^ii^	0.83 (3)	1.89 (3)	2.717 (2)	175 (3)

Symmetry codes: (ii) (i) 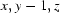; 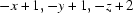. *Cg *is the centroid of the C2–C7 benzene ring.

**Fig. 1.**
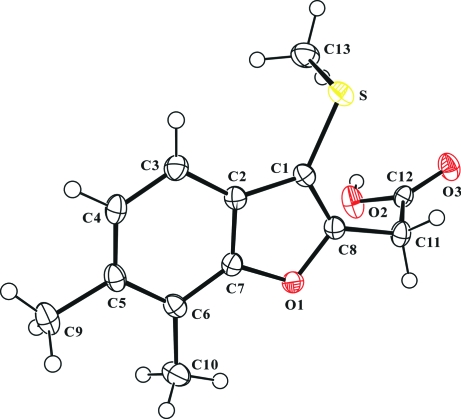
The molecular structure of (I), showing displacement ellipsoids for the non-hydrogen atoms drawn at the 50% probability level.

**Fig. 2.**
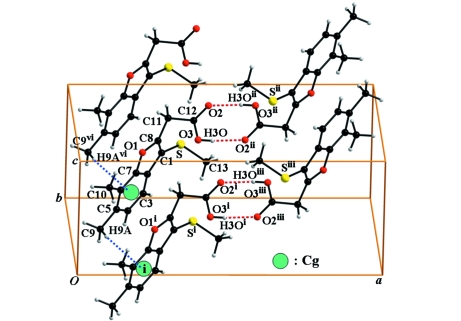
C—H···π interactions and hydrogen bonds (dotted lines) in (I). Cg denotes the ring centroid. [Symmetry code: (i) x, y - 1, z; (ii) -x + 1, -y + 1, -z + 2; (iii) -x + 1, -y, -z + 2; (iv) x, y + 1, z.]

## supplementary crystallographic information

### Comment

This work is related to our communications on the synthesis and structure of 2-(3-methylsulfanyl-1-benzofuran-2-yl)acetic acid derivatives, *viz*. 2-(3-methylsulfanyl-5-phenyl-1-benzofuran-2-yl)acetic acid (Choi *et al.*, 2007) and 2-(5-ethyl-3-methylsulfanyl-1-benzofuran-2-yl)acetic acid (Seo *et al.*, 2007). Here we report the crystal structure of the title compound, (I), 2-(6,7-dimethyl-3-methylsulfanyl-1-benzofuran-2-yl)acetic acid (Fig. 1).

The benzofuran unit is essentially planar, with a mean deviation of 0.003 (1) Å from the least-squares plane defined by the nine constituent atoms. In the crystal structure, the carboxyl groups are involved in intermolecular O—H···O hydrogen bonds (Fig. 2 and Table 1; symmetry code as in Fig. 2), which link the molecules into centrosymmetric dimers. These dimers are further packed into stacks along *a* axis by C—H···π interactions, with a C9—H9A···*Cg*^i^ separation of 2.86 Å (Fig. 2 and Table 1; *Cg* is the centroid of the C2—C7 benzene ring, symmetry code as in Fig. 2).

### Experimental

Ethyl 2-(6,7-dimethyl-3-methylsulfanyl-1-benzofuran-2-yl)acetate (334 mg, 1.20 mmol) was added to a solution of potassium hydroxide (337 mg, 6.0 mmol) in water (20 ml) and methanol (20 ml), and the mixture was refluxed for 5 h, then cooled. Water was added, and the solution was extracted with dichloromethane. The aqueous layer was acidified to pH = 1 with concentrated hydrochloric acid and then extracted with chloroform, dried over magnesium sulfate, filtered and concentrated under vacuum. The residue was purified by column chromatography (ethyl acetate) to afford the title compound as a colorless solid [yield 84%, m.p. 426–427 K; *R*_f_ = 0.63 (ethyl acetate)]. Colorless blocks of (I) were prepared by evaporation of a solution of the title compound in diisopropyl ether at room temperature. Spectroscopic analysis: ^1^H NMR (CDCl_3_, 400 MHz) δ 2.32 (s, 3H), 2.38 (s, 3H), 2.40 (s, 3H), 4.04 (s, 2H), 7.10 (d, J = 7.84 Hz, 1H), 7.36 (d, J = 7.84 Hz, 1H), 9.08 (s, 1H); EI—MS 250 [*M*^+^].

### Refinement

Atom H3O of the hydroxy group was found in a difference Fourier map and refined freely. The other H atoms were positioned geometrically and refined using a riding model, with C—H = 0.95 Å for aromatic H atoms, 0.99 Å for methylene H atoms and 0.98 Å for methyl H atoms, respectively, and with *U*_iso_(H) = 1.2U_eq_(C) for aromatic and methylene H atoms and 1.5U_eq_(C) for methyl H atoms.

### Crystal data

**Table:**

C_13_H_14_O_3_S	*F*_000_ = 528
*M**_r_* = 250.30	*D*_x_ = 1.386 Mg m^−^^3^
Monoclinic, *P*2_1_/*c*	Mo *K*α radiation λ = 0.71073 Å
Hall symbol: -P_2ybc	Cell parameters from 4188 reflections
*a* = 18.050 (2) Å	θ = 3.0–28.3º
*b* = 4.9422 (5) Å	µ = 0.26 mm^−^^1^
*c* = 13.885 (1) Å	*T* = 173 (2) K
β = 104.451 (2)º	Block, colorless
*V* = 1199.4 (2) Å^3^	0.40 × 0.20 × 0.10 mm
*Z* = 4	

### Data collection

**Table:**

Bruker SMART CCD diffractometer	2595 independent reflections
Radiation source: fine-focus sealed tube	2269 reflections with *I* > 2σ(*I*)
Monochromator: graphite	*R*_int_ = 0.035
Detector resolution: 10.0 pixels mm^-1^	θ_max_ = 27.0º
*T* = 173(2) K	θ_min_ = 1.2º
φ and ω scans	*h* = −19→23
Absorption correction: none	*k* = −5→6
6673 measured reflections	*l* = −17→17

### Refinement

**Table:**

Refinement on *F*^2^	Secondary atom site location: difference Fourier map
Least-squares matrix: full	Hydrogen site location: difmap (O-H) and geom (C-H)
*R*[*F*^2^ > 2σ(*F*^2^)] = 0.038	H atoms treated by a mixture of independent and constrained refinement
*wR*(*F*^2^) = 0.119	*w* = 1/[σ^2^(*F*_o_^2^) + (0.0641*P*)^2^ + 0.395*P*] where *P* = (*F*_o_^2^ + 2*F*_c_^2^)/3
*S* = 1.17	(Δ/σ)_max_ < 0.001
2595 reflections	Δρ_max_ = 0.42 e Å^−^^3^
161 parameters	Δρ_min_ = −0.25 e Å^−^^3^
Primary atom site location: structure-invariant direct methods	Extinction correction: none

### Special details

**Table:**

Geometry. All e.s.d.'s (except the e.s.d. in the dihedral angle between two l.s. planes) are estimated using the full covariance matrix. The cell e.s.d.'s are taken into account individually in the estimation of e.s.d.'s in distances, angles and torsion angles; correlations between e.s.d.'s in cell parameters are only used when they are defined by crystal symmetry. An approximate (isotropic) treatment of cell e.s.d.'s is used for estimating e.s.d.'s involving l.s. planes.
Refinement. Refinement of *F*^2^ against ALL reflections. The weighted *R*-factor *wR* and goodness of fit *S* are based on *F*^2^, conventional *R*-factors *R* are based on *F*, with *F* set to zero for negative *F*^2^. The threshold expression of *F*^2^ > σ(*F*^2^) is used only for calculating *R*-factors(gt) *etc*. and is not relevant to the choice of reflections for refinement. *R*-factors based on *F*^2^ are statistically about twice as large as those based on *F*, and *R*- factors based on ALL data will be even larger.

### Fractional atomic coordinates and isotropic or equivalent isotropic displacement parameters (Å^2^)

**Table:**

	*x*	*y*	*z*	*U*_iso_*/*U*_eq_	
S	0.36076 (2)	0.71244 (9)	0.66542 (3)	0.02429 (16)	
O1	0.22123 (6)	0.3898 (3)	0.81259 (8)	0.0204 (3)	
O2	0.40090 (8)	0.3577 (3)	0.94500 (11)	0.0324 (4)	
H2O	0.4453 (16)	0.310 (6)	0.970 (2)	0.049 (8)*	
O3	0.45128 (7)	0.7710 (3)	0.96729 (10)	0.0284 (3)	
C1	0.29079 (9)	0.5385 (3)	0.70833 (12)	0.0189 (4)	
C2	0.23677 (9)	0.3392 (4)	0.65574 (12)	0.0189 (4)	
C3	0.21948 (10)	0.2279 (4)	0.56064 (13)	0.0235 (4)	
H3	0.2463	0.2811	0.5129	0.028*	
C4	0.16167 (10)	0.0365 (4)	0.53846 (13)	0.0252 (4)	
H4	0.1490	−0.0418	0.4739	0.030*	
C5	0.12090 (10)	−0.0469 (4)	0.60716 (13)	0.0229 (4)	
C6	0.13800 (9)	0.0629 (4)	0.70363 (13)	0.0209 (4)	
C7	0.19588 (9)	0.2551 (3)	0.72277 (12)	0.0187 (4)	
C8	0.27891 (9)	0.5601 (4)	0.80010 (12)	0.0192 (4)	
C9	0.05928 (11)	−0.2584 (4)	0.57689 (16)	0.0311 (5)	
H9A	0.0729	−0.4181	0.6195	0.047*	
H9B	0.0543	−0.3097	0.5074	0.047*	
H9C	0.0105	−0.1849	0.5841	0.047*	
C10	0.09738 (11)	−0.0203 (4)	0.78102 (15)	0.0316 (5)	
H10A	0.1197	0.0752	0.8434	0.047*	
H10B	0.1029	−0.2159	0.7921	0.047*	
H10C	0.0430	0.0256	0.7581	0.047*	
C11	0.31794 (10)	0.7259 (4)	0.88784 (13)	0.0221 (4)	
H11A	0.2865	0.7255	0.9370	0.026*	
H11B	0.3218	0.9152	0.8663	0.026*	
C12	0.39713 (10)	0.6221 (4)	0.93705 (12)	0.0197 (4)	
C13	0.43420 (11)	0.4564 (4)	0.68607 (16)	0.0315 (4)	
H13A	0.4529	0.4230	0.7576	0.047*	
H13B	0.4766	0.5183	0.6592	0.047*	
H13C	0.4130	0.2887	0.6526	0.047*	

### Atomic displacement parameters (Å^2^)

**Table:**

	*U*^11^	*U*^22^	*U*^33^	*U*^12^	*U*^13^	*U*^23^
S	0.0226 (3)	0.0218 (3)	0.0288 (3)	−0.00298 (17)	0.00704 (18)	0.00404 (18)
O1	0.0180 (6)	0.0248 (7)	0.0181 (6)	−0.0020 (5)	0.0039 (4)	−0.0033 (5)
O2	0.0245 (7)	0.0204 (7)	0.0432 (8)	0.0008 (5)	−0.0081 (6)	0.0012 (6)
O3	0.0204 (6)	0.0229 (7)	0.0377 (7)	−0.0021 (5)	−0.0005 (5)	−0.0019 (6)
C1	0.0161 (8)	0.0183 (8)	0.0211 (8)	0.0002 (6)	0.0021 (6)	0.0017 (7)
C2	0.0157 (8)	0.0201 (9)	0.0192 (8)	0.0011 (6)	0.0009 (6)	0.0004 (7)
C3	0.0239 (9)	0.0265 (10)	0.0196 (8)	−0.0009 (7)	0.0041 (7)	−0.0021 (7)
C4	0.0266 (9)	0.0251 (10)	0.0206 (8)	−0.0013 (7)	−0.0002 (7)	−0.0060 (7)
C5	0.0179 (8)	0.0190 (9)	0.0277 (9)	0.0006 (6)	−0.0019 (7)	−0.0017 (7)
C6	0.0165 (8)	0.0190 (9)	0.0258 (9)	0.0008 (6)	0.0027 (6)	0.0019 (7)
C7	0.0171 (8)	0.0195 (9)	0.0178 (8)	0.0019 (6)	0.0009 (6)	−0.0014 (7)
C8	0.0153 (8)	0.0186 (9)	0.0218 (8)	0.0012 (6)	0.0011 (6)	−0.0010 (7)
C9	0.0256 (10)	0.0243 (10)	0.0384 (11)	−0.0052 (7)	−0.0012 (8)	−0.0028 (8)
C10	0.0259 (10)	0.0350 (11)	0.0354 (10)	−0.0061 (8)	0.0106 (8)	0.0015 (9)
C11	0.0206 (9)	0.0203 (9)	0.0221 (8)	0.0014 (7)	−0.0006 (7)	−0.0047 (7)
C12	0.0218 (9)	0.0209 (9)	0.0156 (7)	−0.0002 (7)	0.0032 (6)	−0.0027 (7)
C13	0.0258 (10)	0.0310 (11)	0.0402 (11)	0.0012 (8)	0.0127 (8)	0.0018 (9)

### Geometric parameters (Å, °)

**Table:**

S—C1	1.7506 (17)	C5—C9	1.507 (2)
S—C13	1.803 (2)	C6—C7	1.388 (2)
O1—C8	1.383 (2)	C6—C10	1.502 (2)
O1—C7	1.387 (2)	C8—C11	1.491 (2)
O2—C12	1.312 (2)	C9—H9A	0.9800
O2—H2O	0.83 (3)	C9—H9B	0.9800
O3—C12	1.212 (2)	C9—H9C	0.9800
C1—C8	1.348 (2)	C10—H10A	0.9800
C1—C2	1.449 (2)	C10—H10B	0.9800
C2—C7	1.388 (2)	C10—H10C	0.9800
C2—C3	1.392 (2)	C11—C12	1.512 (2)
C3—C4	1.385 (3)	C11—H11A	0.9900
C3—H3	0.9500	C11—H11B	0.9900
C4—C5	1.404 (3)	C13—H13A	0.9800
C4—H4	0.9500	C13—H13B	0.9800
C5—C6	1.407 (2)	C13—H13C	0.9800
			
C1—S—C13	99.52 (9)	C5—C9—H9A	109.5
C8—O1—C7	105.6 (1)	C5—C9—H9B	109.5
C12—O2—H2O	110 (2)	H9A—C9—H9B	109.5
C8—C1—C2	106.5 (2)	C5—C9—H9C	109.5
C8—C1—S	125.5 (1)	H9A—C9—H9C	109.5
C2—C1—S	127.9 (1)	H9B—C9—H9C	109.5
C7—C2—C3	119.2 (2)	C6—C10—H10A	109.5
C7—C2—C1	105.6 (1)	C6—C10—H10B	109.5
C3—C2—C1	135.3 (2)	H10A—C10—H10B	109.5
C4—C3—C2	117.3 (2)	C6—C10—H10C	109.5
C4—C3—H3	121.3	H10A—C10—H10C	109.5
C2—C3—H3	121.3	H10B—C10—H10C	109.5
C3—C4—C5	122.9 (2)	C8—C11—C12	112.5 (1)
C3—C4—H4	118.6	C8—C11—H11A	109.1
C5—C4—H4	118.6	C12—C11—H11A	109.1
C4—C5—C6	120.4 (2)	C8—C11—H11B	109.1
C4—C5—C9	119.3 (2)	C12—C11—H11B	109.1
C6—C5—C9	120.3 (2)	H11A—C11—H11B	107.8
C7—C6—C5	115.0 (2)	O3—C12—O2	123.7 (2)
C7—C6—C10	121.9 (2)	O3—C12—C11	122.7 (2)
C5—C6—C10	123.1 (2)	O2—C12—C11	113.5 (2)
O1—C7—C6	124.4 (2)	S—C13—H13A	109.5
O1—C7—C2	110.4 (1)	S—C13—H13B	109.5
C6—C7—C2	125.3 (2)	H13A—C13—H13B	109.5
C1—C8—O1	111.9 (2)	S—C13—H13C	109.5
C1—C8—C11	131.4 (2)	H13A—C13—H13C	109.5
O1—C8—C11	116.7 (2)	H13B—C13—H13C	109.5
			
C13—S—C1—C8	96.66 (17)	C10—C6—C7—O1	−0.7 (3)
C13—S—C1—C2	−80.90 (17)	C5—C6—C7—C2	−0.8 (3)
C8—C1—C2—C7	−0.11 (19)	C10—C6—C7—C2	178.93 (17)
S—C1—C2—C7	177.82 (13)	C3—C2—C7—O1	−179.69 (15)
C8—C1—C2—C3	179.6 (2)	C1—C2—C7—O1	0.06 (18)
S—C1—C2—C3	−2.5 (3)	C3—C2—C7—C6	0.6 (3)
C7—C2—C3—C4	−0.1 (3)	C1—C2—C7—C6	−179.65 (16)
C1—C2—C3—C4	−179.80 (19)	C2—C1—C8—O1	0.13 (19)
C2—C3—C4—C5	0.0 (3)	S—C1—C8—O1	−177.87 (12)
C3—C4—C5—C6	−0.3 (3)	C2—C1—C8—C11	178.24 (17)
C3—C4—C5—C9	−179.47 (17)	S—C1—C8—C11	0.2 (3)
C4—C5—C6—C7	0.7 (2)	C7—O1—C8—C1	−0.09 (18)
C9—C5—C6—C7	179.84 (16)	C7—O1—C8—C11	−178.51 (14)
C4—C5—C6—C10	−179.11 (17)	C1—C8—C11—C12	−72.5 (2)
C9—C5—C6—C10	0.1 (3)	O1—C8—C11—C12	105.56 (17)
C8—O1—C7—C6	179.73 (16)	C8—C11—C12—O3	138.89 (18)
C8—O1—C7—C2	0.01 (18)	C8—C11—C12—O2	−42.1 (2)
C5—C6—C7—O1	179.48 (15)		

### Hydrogen-bond geometry (Å, °)

**Table:**

*D*—H···*A*	*D*—H	H···*A*	*D*···*A*	*D*—H···*A*
C9—H9A···Cg^i^	0.98	2.86	3.617 (2)	135
O2—H2O···O3^ii^	0.83 (3)	1.89 (3)	2.717 (2)	175 (3)

Symmetry codes: (i) *x*, *y*−1, *z*; (ii) −*x*+1, −*y*+1, −*z*+2.

## References

[bb1] Brandenburg, K. (1998). *DIAMOND* Crystal Impact GbR, Bonn, Germany.

[bb2] Bruker (2001). *SAINT* and *SMART* Bruker AXS Inc., Madison, Wisconsin, USA.

[bb3] Choi, H. D., Seo, P. J., Son, B. W. & Lee, U. (2007). *Acta Cryst.* E**63**, o3468.

[bb4] Farrugia, L. J. (1997). *J. Appl. Cryst.* **30**, 565.

[bb5] Seo, P. J., Choi, H. D., Son, B. W. & Lee, U. (2007). *Acta Cryst.* E**63**, o2048–o2049.

[bb6] Sheldrick, G. M. (2008). *Acta Cryst.* A**64**, 112–122. 10.1107/S0108767307043930 18156677

